# Evaluation of Dried Blood Spots Collected on Filter Papers from Three Manufacturers Stored at Ambient Temperature for Application in HIV-1 Drug Resistance Monitoring

**DOI:** 10.1371/journal.pone.0109060

**Published:** 2014-10-10

**Authors:** Erin K. Rottinghaus, R. Suzanne Beard, Ebi Bile, Mosetsanagape Modukanele, Maruping Maruping, Madisa Mine, John Nkengasong, Chunfu Yang

**Affiliations:** 1 International Laboratory Branch, Division of Global HIV/AIDS, Center for Global Health, Centers for Disease Control and Prevention, Atlanta, Georgia, United States of America; 2 CDC-Botswana, Gaborone, Botswana; 3 Botswana Ministry of Health, Gaborone, Botswana; Fundacion Huesped, Argentina

## Abstract

As more HIV-infected people gain access to antiretroviral therapy (ART), monitoring HIV drug resistance (HIVDR) becomes essential to combat both acquired and transmitted HIVDR. Studies have demonstrated dried blood spots (DBS) are a suitable alternative in HIVDR monitoring using DBS collected on Whatman 903 (W-903). In this study, we sought to evaluate two other commercially available filter papers, Ahlstrom 226 (A-226) and Munktell TFN (M-TFN), for HIVDR genotyping following ambient temperature storage. DBS were prepared from remnant blood specimens collected from 334 ART patients and stored at ambient temperature for a median time of 30 days. HIV-1 viral load was determined using NucliSENS EasyQ® HIV-1 v2.0 RUO test kits prior to genotyping of the protease and reverse transcriptase regions of the HIV-1 *pol* gene using an in-house assay. Among the DBS tested, 26 specimens had a viral load ≥1000 copies/mL in all three types of filter paper and were included in the genotyping analysis. Genotyping efficiencies were similar between DBS collected on W-903 (92.3%), A-226 (88.5%), and M-TFN (92.3%) filter papers (P = 1.00). We identified 50 DR-associated mutations in DBS collected on W-903, 33 in DBS collected on A-226, and 48 in DBS collected on M-TFN, resulting in mutation detection sensitivities of 66.0% for A-226 and 88.0% for M-TFN when compared to W-903. Our data indicate that differences among filter papers may exist at this storage condition and warrant further studies evaluating filter paper type for HIVDR monitoring.

## Introduction

The number of HIV-infected people on antiretroviral therapy (ART) in low- and middle-income countries increased by more than 20% from 2010 to 2011 and continues to increase dramatically every year [1]. As the number of people on therapy rises, there is a profound need for drug resistance (DR) monitoring to combat both acquired and transmitted HIV drug resistance (HIVDR). The standard specimen type for assessing HIVDR is plasma, but multiple studies have been conducted verifying the usefulness of dried blood spots (DBS) as a suitable alternative to plasma [2]–[7]. The use of DBS for HIVDR monitoring is essential in resource-limited countries, as plasma requires timely processing and cold chain for storage and transportation.

Several studies have assessed DBS performance in HIVDR genotyping under various storage temperatures and humidity ranges with variable success (reviewed in [8], [9]). It has been suggested that extended storage of DBS at 4°C [10] or room temperature (RT) [5] make genotyping of larger *pol* gene fragments difficult. This has been found to be particularly true if humidity is not controlled [11], but results still vary from study to study (reviewed in [8], [9]). For instance, Garcia-Lerma *et al*. found that at 37°C with high humidity, DBS specimens were only stable for one to two weeks [12]. However, Bertagnolio *et al*. tested DBS specimens that had been stored at 37°C and with 85% humidity for three months and found an amplification rate of 90% [13]. DBS specimens have proven to be stable at −20°C or below for years in many different studies with consistent outcomes (reviewed in [8], [9]).

Most of the aforementioned studies utilized Whatman 903 filter paper (W-903) to evaluate DBS. As mentioned previously, there have been many studies comparing DBS to plasma, but only one recent study by our laboratory has compared different types of filter papers for HIVDR genotyping [14]. In this study, we evaluated two other commercially available filter paper cards, Munktell TFN (M-TFN) and Ahlstrom grade 226 (A-226) and compared them to Whatman 903 (W-903) filter paper [14]. We found that DBS collected on these two filter paper cards performed similarly to the ones collected on W-903 cards for both HIVDR genotyping and viral load analysis when the DBS cards were stored at −80°C. Storage at −80°C is the gold standard for DBS storage [15], however is not always attainable in many resource-limited settings. We therefore sought to assess the impact of ambient temperature storage on HIV-1 viral load and HIVDR analysis on DBS collected on M-TFN and A-226 filter papers compared to W-903. The current study was part of a project in our laboratory to increase the reservoir of commercially available filter paper types, enhance competition of filter paper suppliers and reduce the cost of filter papers in resource-limited settings for HIVDR monitoring surveys using DBS specimens.

## Materials and Methods

### Specimen Collection and Storage

Specimen collection was described in detail previously [14]. Briefly, DBS specimens were collected from 334 HIV-positive patients who were reported to be on highly active antiretroviral therapy (HAART). DBS specimens were collected from remnant whole blood specimens that were sent to the Nyangabgwe HIV Reference Laboratory in Francistown, Botswana for clinical CD4 monitoring. Blood was stored at ambient temperature (median = 31°C, range = 25–37°C; median humidity = 33%, range humidity = 20–45%) for a median of 1 day (range ≤1–3 days) prior to DBS preparation. No personal (including duration on HAART) or demographic information was collected for this study. DBS specimens were prepared by pipetting 100 µL of whole blood per spot onto Whatman 903 (Whatman plc, Springfield Mill, UK), Ahlstrom grade 226 (Ahlstrom Corporation, Helsinki, Finland), and Munktell TFN (Munktell Inc, Raleigh, NC) filter papers. Filter paper was allowed to dry overnight at ambient temperature (median temperature = 31°C, median humidity = 33%). The next day glassine paper was folded around each DBS card, and 10–25 cards were packaged in a Bitran bag with desiccant packs and a humidity indicator card. Packaged DBS specimens were stored at ambient temperature for a median of 30 days prior to being received at the WHO-designated Specialized Drug Resistance Laboratory at the Centers for Disease Control and Prevention (CDC), Atlanta, GA, U.S. for testing. All specimens were stored at -80°C upon arrival at CDC.

### Ethics Statement

In accordance with United States regulations and international guidelines, the protocol was approved by the Institutional Review Board of the Ministry Health of Botswana. The anonymous testing at CDC was determined as non-human subjects research by the Associate Director for Science at the Center for Global Health, CDC, Atlanta, GA, USA.

### Nucleic Acid Extraction and HIV-1 VL Analysis

One DBS spot (100 µL) was cut out per specimen and placed in 2 mL of NucliSENS® lysis buffer (Biomeriuex, Durham, NC) for 30 min at room temperature with gentle rotation. Nucleic acid was then extracted from all specimens using the NucliSENS® EasyMag® (Biomeriuex, Durham, NC) automated extraction system following the manufacturer's instruction. Nucleic acid was eluted in 25 µL of NucliSENS® Extraction Buffer 3 and stored at −80°C until use. HIV-1 viral load was determined by the NucliSENS EasyQ® automated system using NucliSENS EasyQ® HIV-1 v2.0 RUO test kits (Biomeriuex, Durham, NC) following the manufacturer's instructions. The linear range of this assay is 500–21,000,000 copies/mL when a single DBS spot containing 100 µL of whole blood is used [16].

### HIV-1 Drug-Resistance Genotyping

Genotyping of the protease and reverse transcriptase (RT) regions of the HIV-1 *pol* gene was performed using a broadly sensitive in-house genotyping assay described in detail previously [7], [17]. Briefly, a 1084 base-pair segment of the 5′ region of the *pol* gene was generated by RT-PCR and followed by nested PCR. This fragment was purified, sequenced using the BigDye® Terminator v3.1 Cycle Sequencing Kit (Applied Biosystems, Foster City, CA), and analyzed on the ABI Prism™ 3730 Genetic Analyzer (Applied Biosystems). Specimens that failed to amplify were repeated once with an alternative RT-PCR primer to account for potential mutations in the original primer binding site following the standard practice in our laboratory. The ReCALL software program was used to edit the raw sequences and generate consensus sequences [18]. Phylogenetic analyses were conducted with all the newly obtained sequences along with HIV-1 reference sequences downloaded from the HIV database (http://www.hiv.lanl.gov/content/sequence/NEWALIGN/align.html#ref) to ensure the absence of contamination and confirm clustering of related samples using MEGA [19]. HIV drug-resistance mutations and drug susceptibility profiles were determined using HIVdb and HIValg programs deployed at the Stanford University Drug Resistance Database (Palo Alto, CA). Unique sequences generated in this study were submitted to GenBank under the following accession numbers: KM387674-KM387706.

### Statistical Analysis

Nucleotide sequence identity was calculated using the BioEdit sequence alignment editor [20]. Statistical calculations were performed using GraphPad Prism (version 5.0, GraphPad Software, La Jolla, CA). Fisher's exact test was used to compare the genotyping efficiency and HIVDR mutation frequency of DBS specimens collected on A-226 and M-TFN to the ones collected on W-903 filter paper. Kappa Statistic was used to assess the concordance between the test filter papers (A-226 and M-TFN) and the gold standard (W-903) for HIVDR mutation detection; values were categorized as poor (<0.40), good (0.4 to 0.75), or excellent (>0.75) [21].

## Results

### HIV-1 pol Genotyping Efficiency

Due to viral load variability described previously [14] and the lack of a plasma gold standard control, we limited our genotyping analyses to only those specimens that had a viral load ≥1,000 copies/mL in all three types of filter paper tested. Among the 334 specimens analyzed, we identified 26 specimens that met these criteria. Table 1 illustrates that the overall genotyping efficiencies for these DBS specimens were 88.5% to 92.3% among the three types of filter paper. Although W-903 and M-TFN filter papers had higher genotyping rates than the A-226, there were no statistically significant differences among the filter paper types (p = 1.00). In addition, there were four specimens that had viral load ≥1,000 copies/mL and failed to amplify or genotype in at least one type of the filter papers (Table 2). Of these four specimens: one specimen was not amplified in any type of the filter papers, one was amplified but failed genotyping on W-903 only, one specimen was amplified on W-903 but not the other two filter papers, and one specimen failed amplification on A-226 only (Table 2). Nucleotide sequence identity to W-903 filter paper was similar between A-226 (98.9±0.8) and M-TFN (98.6±1.2) (Table 1).

**Table 1:** Genotyping efficiency and nucleotide sequence identities of 26 DBS specimens with a VL ≥1000 copies/mL and collected on W-903, A-226, and M-TFN filter papers.

	W-903	A-226	M-TFN
**Genotyping Efficiency**	92.3% (24/26)	88.5% (23/26)	92.3% (24/26)
**P-value** *		1.00	1.00
**Nucleotide Identity to W-903 (Mean ± SD^#^)**		98.9±0.8	98.6±1.2

*:Fisher's exact test; ^#^SD: Standard deviation.

**Table 2:** HIV drug resistance mutation Profiles of DBS specimens collected on W-903, A-226, and M-TFN filter papers.

	W-903		A-226		M-TFN	
Specimen #	NRTI	NNRTI	NRTI	NNRTI	NRTI	NNRTI
**10**						**E138AE**
**23**	No PCR Product		No PCR Product		No PCR Product	
35*	D67DN, K70R, M184V, K219KQ	Y181C,	M184V	Y181C	D67DN, K70KR, M184MV, K219KQ,	Y181CY, **H221HY**
**62**	D67N, M184V	L100I, K103N	D67N, M184V	L100I, K103N	D67N, M184V,	L100I, K103N
**64**	D67N, K70R, M184V, T215F, K219Q	V106M, Y181C	D67N, K70R, M184V, T215F, K219Q	V106M, Y181C	D67N, K70R, M184V, T215F, K219Q	V106M, Y181C
**69**	V118I	E138A	V118I	E138A	V118I	E138A
**90**	M184V	K103N	M184V	K103N	M184V	K103N
**155**		K103KN		K103KN		K103KN
**182**		K103N, V106MV		K103N, V106MV		K103N, V106MV
**183**	M184V	Y188L, K238N	M184V	Y188L, K238N	M184V	Y188L, K238N
255*	**M184MV**					
**269**	M41L, M184V, T215Y	Y188L	M41L, M184V, T215Y	Y188L	M41L, M184V, T215Y	Y188L
**280**	D67G, M184V	K103N, P225H	D67G, M184V	K103N	D67DG, M184V, **T215IT**	K103N, P225H
**287**	M184V	V106M, V179D	M184V	V106M, V179D	M184V	V106M, V179D
317*	**D67DN,** **K70KR**, M184V, **K219KQ**	A98G, K103N			M184V	A98G, K103N
**147**	Genotyping Failed					
295*	K65R, **D67N**, Y115F	**V90I**, K103N, V106M	No PCR Product		K65R, Y115F, **K219R**	K103N, V106M
**328**			No PCR Product		No PCR Product	
**Total # Mutations**	50		33		48	

HIV-1 drug resistance genotyping analyses of the *pol* region were performed for all the DBS specimens with a viral load ≥1,000 copies/mL and with all three types of the filter papers using a broadly sensitive genotyping assay (N = 26). Drug resistance mutations against nucleoside reverse transcriptase inhibitors (NRTIs) and non-nucleoside reverse transcriptase inhibitors (NNRTIs) were identified using the HIVdb program, and HIV-1 drug resistance profiles were determined by the HIValg program at the Stanford HIV Drug Resistance Database website. Discordant mutations that were identified in only one type of filter paper are shown in boldface type. Specimens that had a difference in drug susceptibility ratings with one of the filter paper types are indicated by asterisk (*). Eight specimens with no mutations detected in any of the filter paper types were excluded from the table.

### HIV Drug Resistance Mutation Frequency and Profiles

Accurate identification of drug resistance mutations is the most important aspect of an HIVDR monitoring survey. To determine whether filter paper type affected HIVDR mutation profiles and whether identified differences resulted in significant changes to drug susceptibility, we compared the HIVDR mutation profiles between filter paper types for those 26 specimens that had VL ≥1,000 copies/mL in all three types of filter paper. Overall, we identified 50 nucleoside reverse transcriptase inhibitor (NRTI) and non-nucleoside reverse transcriptase inhibitor (NNRTI) mutations in DBS collected on W-903, 33 in specimens collected on A-226, and 48 in DBS collected on M-TFN (Table 2). Out of the 26 specimens analyzed, 18 displayed identical DR mutation profiles between all three types of filter paper with nine having identical HIVDR mutation profiles, eight having no HIVDR mutations detected, and one failing to amplify in all three filter paper types (specimen 23) (Table 2). Four specimens had discordant HIVDR profiles that led to differences in the drug susceptibility rating (Table 2). Two of these discordant specimens were due to mutations identified in W-903 filter paper but not in A-226 or M-TFN; one was the result of mutations identified on W-903 and M-TFN but not on A-226, and the fourth specimen had an H221H/Y mixture that was detectable only in the specimen collected on M-TFN. Two-by-two tables comparing HIVDR mutation detection in A-226 and M-TFN to W-903 were constructed using the International AIDS Society 2011 list of NRTI and NNRTI HIVDR mutation sites [22]. These analyses revealed an overall HIVDR mutation detection sensitivity of 66.0% for A-226 (P = 0.071) and 88.0% for M-TFN (P = 0.917) when compared to DBS collected on W-903 (Table 3). Kappa values illustrated excellent concordance between DBS collected on A-226 (0.79±0.05) and M-TFN (0.89±0.03) compared to W-903 (Table 3).

**Table 3:** Comparison of HIV drug resistance mutations detected in DBS specimens collected on A-226 or M-TFN to W-903.

		W-903				
		**Pos**	**Neg**	**Total**	**Sensitivity (%)**	66.0
**A-226**	**Pos**	33	0	33	**Specificity (%)**	100.0
	**Neg**	17	*756	773	**Kappa ± SD**	0.79±0.05
	**Total**	50	756	806		

		W-903				
		**Pos**	**Neg**	**Total**	**Sensitivity (%)**	88.0
**M-TFN**	**Pos**	44	4	48	**Specificity (%)**	99.5
	**Neg**	6	*752	758	**Kappa ± SD**	0.89±0.03
	**Total**	50	756	806		

*Calculated from 31 possible HIV drug resistance mutations against nucleoside reverse transcriptase inhibitors (NRTIs) and non-nucleoside reverse transcriptase inhibitors (NNRTIs).

## Discussion

The objective of this study was to determine whether DBS specimens collected on W-903, A-226, and M-TFN filter papers and stored at ambient temperature performed similarly for HIV-1 drug resistance genotyping. Genotyping efficiencies were not statistically different between the DBS specimens collected on A-226 (p = 1.00) and M-TFN (p = 1.00) compared to those collected on W-903 (Table 1). Out of 26 specimens analyzed, four had discordant HIVDR profiles that resulted in differences in drug susceptibility between one or two of the filter paper types (Table 2). Of the three types of filter paper, DBS collected on A-226 and stored at ambient temperature appeared to be the least sensitive for HIVDR genotyping (Tables 2-3), although statistical significance was not reached, likely due to the small sample size.

This study was limited by a small sample size and the lack of a true gold standard plasma specimen. This study utilized remnant specimens from CD4 monitoring and the specimens were therefore not available for plasma separation until a median of 1 days (range <1-3 days) after collection. We felt that specimen integrity was compromised due to the duration and temperature (median = 31°C) of storage and opted to exclude these plasma specimens from the study. To compensate for the absence of a gold standard specimen, we limited our analysis to those specimens that had a detectable VL (defined as viral load ≥1,000 copies/mL) in all three types of filter paper to help standardize the analyses. In doing so, we were only able to analyze 26 specimens and thus cannot make definitive recommendations regarding the performance of filter papers for HIV drug resistance analysis.

The genotyping efficiencies illustrated in Table 1 were similar for all three types of filter paper, indicating that there were no differences in maintaining HIV-1 RNA integrity between the filter papers. The efficiencies achieved in this study of 92.3% for W-903, 88.5% for A-226 and 92.3% for M-TFN were in agreement with previous studies using this particular genotyping assay on DBS specimens. These previous studies demonstrated amplification/genotyping efficiencies of 77.8% [14], 80.6% [14], 89.5% [2], 93.3% [14], 95.8% [7] and 100% [6]. Despite similar genotyping efficiencies for all three types of filter paper, we observed a bias in the detection of drug resistance mutations in DBS collected on A-226 filter paper. DBS collected on A-226 detected 33 HIVDR mutations, compared to 50 from W-903 and 48 from M-TFN (Table 2). Furthermore, there was an even distribution of false-positive and false-negative mutations detected in M-TFN compared to W-903 (Table 3). This distribution was skewed in the analysis of A-226 with zero false-positive and 17 false-negative mutations detected compared to W-903 (Table 3). This bias was not evident in a previous study comparing DBS collected on the three types of filter paper and stored at -80°C [14]. These data indicate that in this study, DBS collected on A-226 filter paper and stored at ambient temperature did not perform as well as DBS collected on W-903 and M-TFN for HIVDR genotyping analysis.

The limitations of this study prevent us from making overarching conclusions regarding the similarity or differences of DBS collected on A-226, M-TFN, and W-903 filter papers and stored at ambient temperature. Our data do however indicate that differences may exist at this storage condition and warrant further studies comparing different types of filter paper. Such studies are critical for expanding the availability of filter paper for DBS collection for HIVDR monitoring surveys and will likely lead to decreased costs for the future HIVDR monitoring surveys.

## References

[1] UNAIDS (2012) Global report: UNAIDS report on the global AIDS epidemic 2012. Joint United Nations Programme on HIV/AIDS (UNAIDS).

[2] Inzaule S, Yang C, Kasembeli A, Nafisa L, Okonji J, et al (2013) Field evaluation of a broadly sensitive HIV-1 in-house genotyping assay for use with both plasma and dried blood spot specimens in a resource-limited country. J Clin Microbiol 51: 529–539. 23224100 10.1128/JCM.02347-12 PMC3553877

[3] Johannessen A, Garrido C, Zahonero N, Sandvik L, Naman E, et al (2009) Dried blood spots perform well in viral load monitoring of patients who receive antiretroviral treatment in rural Tanzania. Clin Infect Dis 49: 976–981. 19663598 10.1086/605502

[4] Masciotra S, Garrido C, Youngpairoj AS, McNulty A, Zahonero N, et al (2007) High concordance between HIV-1 drug resistance genotypes generated from plasma and dried blood spots in antiretroviral-experienced patients. AIDS 21: 2503–2511. 18025887 10.1097/QAD.0b013e3281c618db

[5] McNulty A, Jennings C, Bennett D, Fitzgibbon J, Bremer JW, et al (2007) Evaluation of dried blood spots for human immunodeficiency virus type 1 drug resistance testing. J Clin Microbiol 45: 517–521. 17166967 10.1128/JCM.02016-06 PMC1829056

[6] Rottinghaus EK, Ugbena R, Diallo K, Bassey O, Azeez A, et al (2012) Dried Blood Spot Specimens Are a Suitable Alternative Sample Type for HIV-1 Viral Load Measurement and Drug Resistance Genotyping in Patients Receiving First-Line Antiretroviral Therapy. Clin Infect Dis 54: 1187–1195. 22412066 10.1093/cid/cis015 PMC11528918

[7] Zhou Z, Wagar N, DeVos JR, Rottinghaus E, Diallo K, et al. (2011) Optimization of a low cost and broadly sensitive genotyping assay for HIV-1 drug resistance surveillance and monitoring in resource-limited settings. PLoS One 6: e28184. 10.1371/journal.pone.0028184 PMC3223235 22132237

[8] Bertagnolio S, Parkin NT, Jordan M, Brooks J, Garcia-Lerma JG (2010) Dried blood spots for HIV-1 drug resistance and viral load testing: A review of current knowledge and WHO efforts for global HIV drug resistance surveillance. AIDS Rev 12: 195–208. 21179184

[9] Hamers RL, Smit PW, Stevens W, Schuurman R, Rinke de Wit TF (2009) Dried fluid spots for HIV type-1 viral load and resistance genotyping: a systematic review. Antivir Ther 14: 619–629. 19704164

[10] Youngpairoj AS, Masciotra S, Garrido C, Zahonero N, de Mendoza C, et al (2008) HIV-1 drug resistance genotyping from dried blood spots stored for 1 year at 4 degrees C. J Antimicrob Chemother. 61: 1217–1220. 10.1093/jac/dkn100 PMC2386080 18344550

[11] Garrido C, Zahonero N, Fernandes D, Serrano D, Silva AR, et al (2008) Subtype variability, virological response and drug resistance assessed on dried blood spots collected from HIV patients on antiretroviral therapy in Angola. J Antimicrob Chemother 61: 694–698. 18218644 10.1093/jac/dkm515

[12] Garcia-Lerma JG, McNulty A, Jennings C, Huang D, Heneine W, et al (2009) Rapid decline in the efficiency of HIV drug resistance genotyping from dried blood spots (DBS) and dried plasma spots (DPS) stored at 37 degrees C and high humidity. J Antimicrob Chemother 64: 33–36. 19403653 10.1093/jac/dkp150 PMC2692501

[13] Bertagnolio S, Soto-Ramirez L, Pilon R, Rodriguez R, Viveros M, et al (2007) HIV-1 drug resistance surveillance using dried whole blood spots. Antivir Ther 12: 107–113. 17503754

[14] Rottinghaus E, Bile E, Modukanele M, Maruping M, Mine M, et al (2013) Comparison of Ahlstrom grade 226, Munktell TFN, and Whatman 903 filter papers for dried blood spot specimen collection and subsequent HIV-1 load and drug resistance genotyping analysis. J Clin Microbiol 51: 55–60. 23077127 10.1128/JCM.02002-12 PMC3536251

[15] WHO (2010) WHO Manual for HIV Drug Resistance Testing Using Dried Blood Spot Specimens.

[16] Biomerieux NucliSens EasyQ HIV-1 v2.0 RUO Package Insert.

[17] Yang C, McNulty A, Diallo K, Zhang J, Titanji B, et al (2010) Development and application of a broadly sensitive dried-blood-spot-based genotyping assay for global surveillance of HIV-1 drug resistance. J Clin Microbiol 48: 3158–3164. 20660209 10.1128/JCM.00564-10 PMC2937690

[18] Woods CK, Brumme CJ, Liu TF, Chui CK, Chu AL, et al (2012) Automating HIV drug resistance genotyping with RECall, a freely accessible sequence analysis tool. J Clin Microbiol 50: 1936–1942. 22403431 10.1128/JCM.06689-11 PMC3372133

[19] Tamura K, Peterson D, Peterson N, Stecher G, Nei M, et al (2011) MEGA5: Molecular Evolutionary Genetics Analysis using Maximum Likelihood, Evolutionary Distance, and Maximum Parsimony Methods. Molecular Biology and Evolution 28: 2731–2739. 21546353 10.1093/molbev/msr121 PMC3203626

[20] Hall TA (1999) A user-friendly biological sequence alignment editor and analysis program for window96/98/NT. Nucleic Acids Symposium Series: 95–98.

[21] Fleiss JL, editor (1981) Statistical Methods for Rates and Proportions. 2nd ed. New York: John Wiley.

[22] Johnson VA, Calvez V, Gunthard HF, Paredes R, Pillay D, et al (2011) 2011 update of the drug resistance mutations in HIV-1. Top Antivir Med 19: 156–164. 22156218 PMC6148877

